# Statistical fragility of reporting hemidiaphragmatic paralysis after brachial plexus blocks in randomized controlled trials: a systematic review

**DOI:** 10.1007/s44254-023-00006-6

**Published:** 2023-04-24

**Authors:** Quehua Luo, Yang Liu, Yi Zhu, Zhipeng Wang, Junyi Zheng, Weifeng Yao

**Affiliations:** 1 Department of Anesthesiology, Guangdong Provincial People’s Hospital (Guangdong Academy of Medical Sciences), Southern Medical University, Guangzhou, 510080, China; 2 Department of Anesthesiology, Sichuan Provincial People’s Hospital, University of Electronic Science and Technology of China, Chengdu, 610072 China; 3 Department of Anesthesiology, the First Affiliated Hospital of Guangzhou University of Chinese Medicine, Guangzhou, 510405 China; 4 Department of Anesthesiology, the Third Affiliated Hospital of Sun Yat-Sen University, Guangzhou, 510630 China

**Keywords:** Fragility index, Hemidiaphragmatic paralysis, Brachial plexus block, Randomized controlled trials

## Abstract

To characterize the fragility index (FI) of statistically significant results reported in randomized controlled trials (RCTs) investigating the incidence of hemidiaphragmatic paralysis (HDP) after brachial plexus blocks. A systematic review of RCTs retrieved from the PubMed-Medline, Embase, and Web of Science electronic databases was conducted. All alternative RCTs published between January 2012 and October 2022 were identified. Only RCTs with two parallel arms designs, and reporting HDP as the primary outcome, statistical significance, and superiority results were selected. The FI was calculated according to Fisher’s exact test using previously described methods. In addition, the risk of bias was evaluated using the Cochrane Risk-of-Bias tool for randomized trials. The 23 RCTs that fulfilled the inclusion criteria had a median FI of 4 (interquartile range [IQR]2–8) and a median Fragility Quotient of 0.077 (IQR 0.038- 0.129). However, in 13 (56.5%) trials, the calculated FI value was ≤ 4. In 3/23 (13.0%) trials, the number of patients who dropped-out exceeded the FI value. Most trials (91.3%) had an overall low risk of bias. This systematic review revealed that the statistical results of RCTs investing HDP after brachial plexus blocks have tended to be fragile in the past decade. The FI should be an important aid in the interpretation of clinical results in combination with the P-value, particularly when statistically significant results are dependent on a small number of events. Future RCTs with larger sample sizes are needed to obtain more robust results in this field.

A *P*-value < 0.05 is typically considered to confirm a positive outcome and reject the null hypothesis in randomized controlled trials (RCTs). Statistically significant findings are likely to be adopted and may influence clinical practice because they represent the highest level of evidence for optimizing patient care [1]. However, positive results interpreted from *P*-values alone have intrinsic limitations and are easily overemphasized in terms of repeatability and statistical reliability [2]. To address this concern, Walsh et al. [3] first proposed the concept of the Fragility Index (FI) based on the methodology, which could serve as a supplemental metric for interpreting clinical results. FI is defined as the minimum number of patients (or events) required to increase the *P*-value to ≥ 0.05. This is performed by converting “non-event” to “events” in one of two groups or vice versa, one patient at a time, until reversion of significance is obtained after recalculating a two-sided Fisher's exact test. For example, a trial with an FI of 3 means that only three patients reverted to the opposite outcome status, it would lead to a loss of statistical significance. A large FI indicated a robust outcome; conversely, a small FI suggests fragile or less robust outcomes. Therefore, the FI reflects the robustness of statistically significant results rather than replacing the P-value. In other words, the lower the FI score(s), the more “fragile” a trial's results. As a derived parameter, the Fragility Quotient (FQ) is calculated by dividing the FI by the total sample size, which provides a similar metric to evaluate fragility.

To date, brachial plexus blocks are the most common regional anesthetic techniques for surgical anesthesia and perioperative analgesia in patients undergoing upper limb surgery [4]. Due to the adjacent position of the trajectory of phrenic nerve palsy and the interscalene groove, hemidiaphragmatic paralysis (HDP) has traditionally been an inevitable consequence of interscalene brachial plexus block [5]. Furthermore, the incidence of HDP is reported to be 45-70% for supraclavicular blocks and 5%-15% for infraclavicular blocks [6–8], probably because of retrograde spread of the local anesthetic to the phrenic nerve. Although generally well-tolerated by healthy patients, HDP may become a critical concern for patients with moderate to severe impairment of lung function. Nevertheless, the incidence of HDP varies according to the site of injection, the new or modified technique used, and the volume/concentration/form of the local anesthetic injected. It has become a hot topic in the anesthetic management of proximal upper limb surgery (e.g., arthroscopic shoulder surgery) [9]. To date, However, no studies have examined FI in RCTs of HDP after brachial plexus blocks. This is important because salient results are usually used to develop recommendations for diaphragm-sparing nerve blocks in clinical anesthesia practice, especially in high-risk patients.

Our primary aim was to evaluate the robustness of statistically significant results reported in anesthesiology RCTs investigating HDP after brachial plexus blocks. Previous studies in the fields of anesthesiology [10, 11], coronavirus disease 2019 [12], and critical care [13] indicate that FI scores are generally low and that it is common for the number of patients lost to follow-up to exceed the FI. Because RCTs in anesthesiology are frequently small, and often have relatively small sample sizes, we hypothesized that statistically significant results are also comparably fragile compared with those in other medical specialties. To test this hypothesis, we systematically reviewed relevant RCTs to calculate the FI scores for the primary outcome and measured HDP after brachial plexus block.

## Methods

### Search strategy and selection of studies

A systematic search of the PubMed-Medline, Excerpta Medica database (Embase), and Web of Science electronic databases was performed to identify all relevant RCTs published between January 1, 2012, and August 15, 2022. This systematic review was designed and is reported in accordance with the Preferred Reporting Items for Systematic Reviews and Meta-analyses (i.e., “PRISMA”) statement [14]. A search update was performed during the review process on October 8, 2022.

A brief explanation of the “PICO” scheme for the search strategy is as follows: P (Problem) was initially defined as adult patients included in RCTs published in the last decade; I (intervention) was defined as brachial plexus blocks, searching for the terms interscalene brachial plexus block OR interscalene block OR interscal* OR supraclavicular brachial plexus block OR supraclavicular block OR supraclav* OR infraclavicular brachial plexus block OR infraclavicular block OR infraclav*; C (comparison/control) was defined as other nerve blocks and injection techniques, searching for the terms nerve block, OR phrenic-sparing OR diaphragm-sparing OR local anesthetic OR injection technique OR injection; and O (outcome) was initially defined as the incidence of HDP, searching for the terms hemidiaphragmatic paresis, diaphragmic paralysis, phrenic nerve, or phrenic nerve palsy.

Two reviewers (QHL and YL) independently screened the titles and abstracts of the retrieved articles. A full-text review was performed for all included studies. Furthermore, references from selected articles, systematic reviews, and meta-analyses were also screened for potentially relevant studies. All discrepancies were resolved by consensus through a discussion groups (QHL, YL and WFY). Potentially eligible studies were screened using the following pre-specified criteria: 1:1 parallel, two-arm RCTs; reporting the incidence of HDP as the primary outcome; and reporting statistically significant results for the primary outcome. Non-inferiority trials and RCTs with cluster or cross-over designs were excluded, as were case reports, case series, technique articles, study protocols, preprint articles, and conference abstracts.

### Data extraction and analysis

A series of data were collected from each RCT, including sample size, number of patients who dropped out, number of patients who experienced HDP, primary outcome, type of comparison, reported P-value, journal name, year of publication, and 2021 Journal Impact Factor. Any unreported information was recorded as “not reported”. Two reviewers (QHL and YL) extracted the data independently and in duplicates using a tailored spreadsheet form (Excel, Microsoft Corporation, Redmond, WA, USA). All extracted data were verified by two other reviewers (YZ and ZPW). The risk of bias in each trial was assessed using the Cochrane Risk-of-bias tool for randomized trials (RoB 2) [15], which would provide a level of risk assessment in Domain 5 based on the answers, including “Randomization process”, “Deviations from intended interventions”, “Missing outcome data”, “Measurements of outcome”, and “Selection of the reported results”. The final judgment of the overall risk of bias was defined as “High” or “Low” risk of bias, or “Some concerns”.

For all selected studies, HDP reported as the primary outcome was chosen for calculating FI. This was determined using a free online FI calculator (http://clincalc.com/Stats/FragilityIndex.aspx), in which was embedded a two-sided Fisher’s exact test combined with stepwise mathematical deduction as described by Walsh et al. [3]. In essence, the process of calculating the FI describes how many patients would be required to make a study’s results not statistically significant (i.e., *P* ≥ 0.05) by converting the number of patients required in one group (control or experimental) from a “non-event” to an “event” status. An FI score of 0 indicated that it was not necessary to revert a patient from a non-event to an event in any group to eliminate statistical differences. In addition, considering the bias from the sample size, the FQ was calculated by dividing the FI by the sample size.

To summarize the characteristics of the included studies, continuous variables are reported as median with corresponding inter-quartile range (IQR), while categorical variables are expressed as count and percentage. The FI, number of drop-outs, and FI minus the number of drop-outs were plotted in histogram form. All statistical analyses were performed using SPSS version 21 (IBM Corp., Armonk, NY, USA).

## Results

### Study selection

**Fig. 1:**
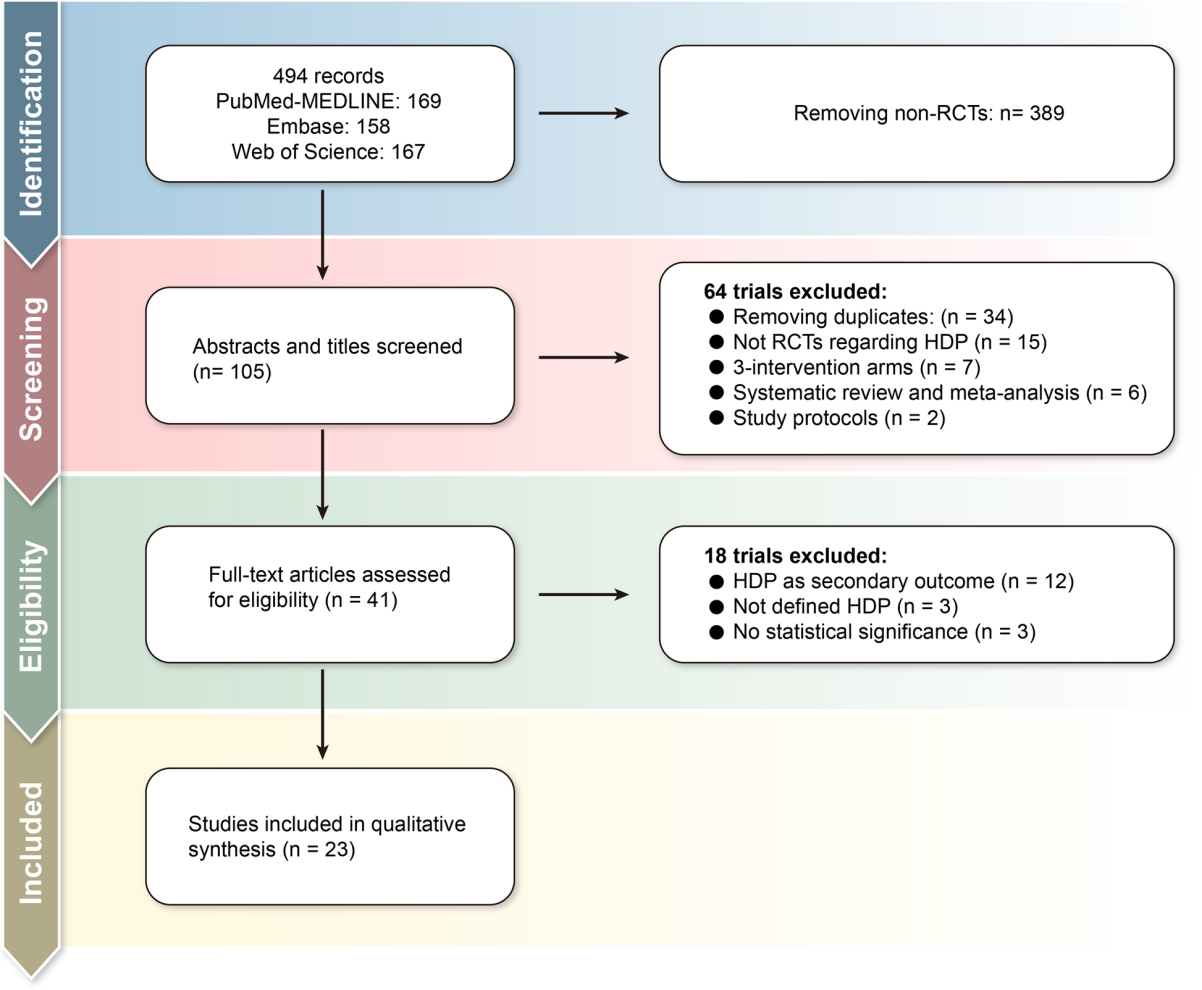
Flowchart of the systematic search. RCTs, randomized controlled trials; HDP, hemidiaphragmatic paralysis

After a comprehensive literature search, 494 trials were retrieved for initial screening. After removing non-RCTs, 105 titles and abstracts were screened for eligibility, of which 64 trials were excluded. Most of the potential trials were eliminated either for duplication in the three databases or for non-specific RCTs addressing the subject of HDP. After screening the remaining 41 full-text articles, 18 were excluded. Most potential trials were eliminated because they reported HDP as a secondary outcome. The remaining 23 trials were included for FI analysis. Detailed of the study inclusion process is illustrated in Fig. 1.

### Study characteristics

Characteristics of the included trials are summarized in Table 1. Of the 23 RCTs, 20 (87.0%) were published in anesthesiology journals. Nine (39.1%) studies addressed the effects of interscalene block on the incidence of HDP, 8 (34.8%) investigated block techniques at the level of the clavicle (e.g., supraclavicular block, costoclavicular block), and 6 (26.1%) compared different techniques. The median sample size was 60 (IQR 40- 74) participants, with a median impact factor of 5.56 (IQR 4.18–8.99). Almost all trials had P-value ≤ 0.01. Risk of bias evaluated according to the RoB 2 tool indicated that most trials (21 of 23) had an overall low risk of bias.

**Table 1:** Study characteristics of the selected RCTs (*n* = 23)

Trails	Name of journal and Impact factor	Risk of bias*	Type of comparison and Reported *P*-value	Sample size	Drop out^#^	Primary outcome and Number of patients	FI score	FQ score
Oliver-Fornies et al., [28] 2022	Anaesthesia 12.893	L	10 mL *vs* 20 mL levo-bupi ISB *P* < *0.001*	48	0	HDP at 4 h after block; 6 *vs* 23	12	0.25
Sun et al., [37] 2022	Can J Anesth. 6.713	L	CISB *vs* HT-ESPB*P* < *0.001*	30	2	HDP in the PACU; 14 *vs* 0	9	0.3
Zhou et al., [36] 2022	Anesth Analg. 6.627	L	ISB *vs* CPB *P* < *0.001*	40	0	HDP at 30 min after block; 10 *vs* 0	4	0.1
Berg et al., [31] 2022	Anesthesiology 8.986	L	Bupi *vs* liposomal Bupi ISB *P* = *0.007*	26	4	HDP at 24 h; 0 *vs* 5	1	0.038
Kim et al., [27] 2021	Pain Physician 4.396	L	Conventional *vs* LVISB *P* = *0.004*	52	0	HDP after block completion; 24 *vs* 14	4	0.077
Kim et al., [24] 2021	Anesth Analg. 6.627	L	15 mL *vs* 5 mL STB *P* < *0.001*	70	0	HDP at 30 min after block; 23 *vs* 5	9	0.129
Hong et al., [6] 2021	Sci Rep. 4.996	L	CCB *vs* SCB *P* = *0.002*	80	5	HDP in the PACU; 4 *vs* 19	5	0.063
Srinivasan et al., [38] 2021	Indian J Anaesth. /	L	Conventional *vs* Saline ISB *P* = *0.002*	36	1	HDP in the PACU; 16 *vs* 8	4	0.111
Georgiadis et al., [7] 2021	Eur J Anaesthesiol. 4.183	L	SCB *vs* RCB *P* = *0.001*	40	0	HDP at 30 min after block; 14 *vs* 3	4	0.1
Zhang et al., [30] 2020	Ann Palliat Med. 1.925	L	20 mL *vs* 30 mL SCB *P* < *0.05*	103	3	HDP at 30 min after block; 26 *vs* 39	3	0.029
Ferré et al., [32] 2020	Anaesthesia 12.893	L	Anterior *vs* Posterior SSnB *P* < *0.001*	84	1	HDP at 30 min after block; 17 *vs* 1	7	0.083
Sivashanmugam et al., [8] 2019	Eur J Anaesthesiol. 4.183	L	SCB *vs* CCB *P* = *0.008*	40	0	HDP at 30 min after block; 9 *vs* 1	2	0.05
Kim et al., [33] 2019	Anesthesiology 8.986	L	ISB *vs* STB *P* < *0.001*	126	1	HDP in the PACU; 45 *vs* 3	31	0.246
Taha et al., [39] 2019	Acta Anaesthesiol Scand. 2.274	L	LVISB *vs* ISO block *P* < *0.001*	73	1	HDP in the PACU; 32 *vs* 2	23	0.315
Bao et al., [26] 2019	Reg Anesth Pain Med. 5.564	L	20 mL *vs* 30 mL SCB *P* = *0.03*	74	6	HDP at 30 min after block; 14 *vs* 23	0	0
Ayyanagouda et al., [40] 2019	Indian J Anaesth. /	L	IF- vs EF-ISB *P* < *0.001*	60	1	HDP at 30 min after block; 14 *vs* 5	1	0.017
Kang et al., [41] 2018	Reg Anesth Pain Med. 5.564	L	IF- *vs* EF- SCB *P* = *0.019*	36	0	HDP at 30 min after block; 13 *vs* 5	2	0.056
Albrecht et al., [42] 2017	Brit J Anaesth. 11.719	L	IF- *vs* EF- CISB *P* = *0.01*	70	2	HDP on postoperative day 1; 14 *vs* 5	1	0.014
Wiesmann et al., [34] 2016	Acta Anaesthesiol Scand. 2.274	S	CISB *vs* CSCB *P* = *0.002*	120	6	HDP in the PACU; 46 *vs* 32	6	0.05
Ghodki et al., [43] 2016	J Anaesthesiol Clin Pharmacol. /	S	NS- vs US- ISB *P* < *0.0001*	60	0	HDP in the PACU; 12 *vs* 0	5	0.083
Palhais et al., [44] 2016	Brit J Anaesth. 11.719	L	IF- *vs* EF- ISB *P* < *0.001*	40	1	HDP at 30 min after block; 18 *vs* 4	8	0.2
Petrar et al., [35] 2015	Reg Anesth Pain Med. 5.564	L	SCB *vs* ISB *P* = *0.001*	64	0	HDP at 30 min after block; 14 *vs* 4	2	0.031
Thackeray et al., [29] 2013	J Shoulder Elbow Surg. 3.507	L	0.125% *vs* 0.25% Bupi CISB *P* = *0.008*	30	1	HDP in the PACU; 3 *vs* 11	2	0.067

^*^Risk of bias: The overall risk of each trial was evaluated by the Cochrane risk-of-bias tool for randomized trials (RoB 2). L, Low risk; S, Some concerns; H, High risk. ^#^Drop out: This refers to the cases in which the subject could not complete all the study process after entering the clinical trial, for example, lost to follow-up

*RCTs *Randomized clinical trials, *ISB *Interscalene block, *CISB* Continuous interscalene block, *IF * Intrafascial, *EF * Extrafascial, *HT-ESPB * High-thoracic erector spinae plane block, *CPB* Cervical plexus block, *Bupi *Bupivacaine, *SSnB *Suprascapular nerve block, *STB *Superior trunk block, *HDP *Hemidiaphragmatic paralysis, *RCB *Retroclavicular block, *SCB * Supraclavicular block, *CSCB *Continuous supraclavicular block, *CCB *Costoclavicular block, *LVISB *Low volume interscalene block, *ISO *Infraclavicular‐subomohyoid block, *US *Ultrasound, *NS *Nerve stimulator,  *PACU *Post anesthesia care unit, *FI * Fragility index,  *FQ * Fragility quotient

### The FI and the number of patients who dropped-out

**Fig. 2:**
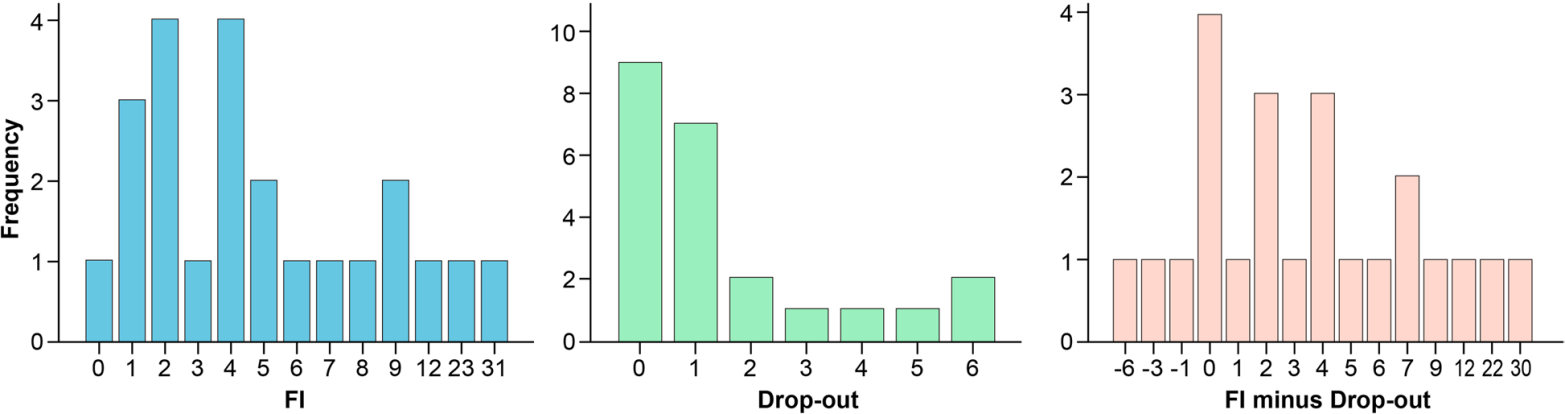
Distribution of FI, the number of patients Drop-out, and FI minus the number of patients Drop-out. When a trial’s *P*-value became non-significant without “converting” a patient from a non-event to an event, the FI is reported as “0”. FI, Fragility Index

The FI values for each study are summarized in Table 1, and the distribution of FI, drop-outs, and “FI minus the number of patients drop-outs” is presented in Fig. 2. The median FI and FQ values for the 23 trials were 4 (IQR 2–8) and 0.077 (IQR 0.038–0.129), respectively. Overall, in 13 of the 23 (56.5%) trials, the calculated FI values were ≤ 4; only one trial had an FI and FQ of 0. The median drop-out rate was 1(range, 0–2). In 3 of 23 (13.0%) trials, the number of patients who dropped out exceeded the FI. The median FI minus the number of patients who dropped out was 3 (IQR 0–7), with 4 of 23 (17.4%) trials having an “FI minus the number of patients who dropped out” of 0.

## Discussion

Results of the present systematic review yielded a median FI of 4, which revealed that ≤ 4 in 50% of primary outcomes from RCTs on investigating HDP were published between 2012 and 2022. This result means that for one-half of the included trials, adding the primary outcome status of 4 subjects to the opposite treatment arm would change the result from statistically significant to non-significant. The results of this review are consistent with those of previously published reviews calculating FI ≤ 4 in anesthesiology, as well as other medical specialties (e.g., gynecological surgery [16], spine surgery [17], and colorectal surgery [18]). In systematic review by Mazzinari et al*.* [10], the median FI was 4 (IQR 2–17) and 3 (IQR 2–7) in trials published in general medicine (35 RCTs) and anesthesiology journals (104 RCTs), respectively. In another systematic review by Bertaggia et al*.* [11], the median FI was 2 (IQR 1–3) after identifying and analyzing 46 trials with 37,347 subjects. The large proportion of similar results is likely attributable to the relatively small sample size. According to previous systematic reviews, a high FI appeared to be associated with sample size and P-value (or effect size) [1, 12, 19], which was also found in 2 studies (Table 1). This suggests that there is room for improvement in the design of RCTs investigating HDP, such as considering increasing the sample size or improving statistical power [20, 21].

In clinical studies, the sample size was calculated a priori, more specifically, at the beginning of the study design [22]. In the field of HDP, calculating the sample size for most studies is based on differences in incidence. The calculation programs can be manipulated by four main variables: alpha (α), beta (β), and assumed rates for both groups [23]. In theory, when a two-tailed α of 0.025 is selected or increasing power (1-β, from 0.8 to 0.9–0.97.5) in varying degrees can inflate the sample size. However, a lack of power analysis or calculation of effect size was ubiquitous in the selected studies. A priori power analysis can be very helpful when designing a primary outcome with wide variation between groups. For example, to demonstrate the sample size calculation process described by Kim et al*. * [24], we performed an a priori power analysis using the Power and Sample size free online calculator (http://powerandsamplesize.com/Calculators/). According to this assumption (expected reduction in HDP incidence ≥ 50%), the hazard ratio or effect size is assumed to be 0.5. We selected “time-to-event” methods for calculating sample size, alpha is defined as 0.05 and the desired power as 0.9, this increases to a sample size of 115 versus 62 in the original article. Therefore, a priori power analysis may be required when designing a trial with a small sample size. However, a small FI does not necessarily imply that the study is trustworthy. Its role is to aid in the interpretation of clinical results and help readers look beyond a statistically significant P-value. Furthermore, a novel algorithm as described by Baer et al*.* [25], was proposed to extend a priori power analysis to simultaneously design for both P-value based test and fragility index-based tests. Therefore, further trials focused on HDP using the FI-based approach for calculating sample size not only resulted in the desired power but also indicated a robust outcome at the design stage.

In the past decade, the incidence of HDP following different brachial plexus blocks has gradually attracted significant interest in clinical anesthesia practice [5, 9]. This is of particular importance when performing anesthesia in moderate-to-high-risk patients with impaired lung function, particularly in ambulatory surgery settings. Of the 23 RCTs, all mainly focused on reducing HDP after brachial plexus blocks, for example, low volume and/or low concentration of local anesthetic [24, 26–30], long-acting local anesthetic [31], distal/selective brachial plexus blocks (e.g., supraclavicular block, superior trunk block) [6–8, 32–35], or needle technique innovation (e.g., extrafascial versus intrafascial injection) [36–44]. However, there is a discrepancy in the definition of HDP among these studies, as well as in the time points for evaluation. In most studies, diaphragmatic paralysis was assessed according to the three-level method described by Renes et al*.* [45]; more specifically, no paralysis 0% to 25% (percent change from the baseline of diaphragmatic excursion according to M-mode ultrasound), partial paralysis 25% to 75%, and complete paralysis 75% to 100%, or the occurrence of paradoxical movement. In other studies, the binary method of occurrence or non-occurrence was used; the cut-off point is that diaphragmatic excursion has decreased by at least 50% or not achieved [8, 26] or self-definition [27, 39]. In addition, the most common time point for evaluation was 30 min after block completion or in the post-anesthesia care unit (PACU) (Table 1). These differences may affect the incidence of HDP and the results of the comparison between different studies. Future studies should use a consistent trial design, and FI should be reported when statistically significant results are revealed. Consequently, readers may gain a more in-depth interpretation of the results by relying on the *P*-value, 95% confidence interval and FI.

In addition, 3 of the 23 trials reported that the number of patients dropped was greater than the calculated FI; 4 of the 23 trials reported that the number of patients dropped equal to the calculated FI. In terms of interventions limited to the perioperative period, we chose the number of patients who dropped out instead of being lost to follow-up, which was more consistent with clinical anesthesia practice. This was defined as the number of patients in whom the status of the study process failed to receive a predetermined block, active withdrawal, or loss to follow-up before completing the primary outcome. The number of patients who dropped out could cause bias when assessing the occurrence of the primary outcome [46]. For example, in a trial with an FI of 3, 6 patients failed to receive a predetermined block or completed the primary outcome; the statistical difference between groups would change to non-significant if ≥ 3 of these patients would have experienced HDP. In other words, if the FI is lower than the number of patients who dropped out, the statistical significance of the reported HDP of a trial could be even more “fragile”. Therefore, it is crucial to realize that if a trial has a high FI, but with a number of patients who dropped out greater than FI needs to be interpreted with caution [1–3]. Furthermore, our study found that the overall risk of bias among the included trials was low, except for two trials. The two RCTs by Wiesmann et al*.* [34] and Ghodki et al*.* [43] in 2016 were assessed with the final judgment of “Some concerns”. The major concerns stemmed from the single-blinded method and the lack of randomization concealment.

The present systematic review has several limitations. First, the total number of selected studies was not exhaustive because the analysis was restricted to trials with two parallel arm designs, defined HDP as the primary outcome, and reported a significant result. The reason for our choice is that identifying the primary and secondary outcomes is the first step in designing and conducting a superiority trial, which is also the basis for sample size calculation. Consequently, some studies were excluded because they defined HDP as a secondary outcome (*n* = 12), had ≥ 2 parallel arms (*n* = 7), and reported no statistical significance (*n* = 3). Second, FI itself has inherent flaws, which can only be used for statistically significant binary outcomes. None of the other binary or continuous outcomes were included in this systematic review. Recently, Caldwell et al*.* [47] proposed a new algorithm, called the Continuous Fragility Index, that can extend fragility to continuous outcomes and provide a method for simulating raw data. The Continuous Fragility Index can be applied to continuous variables such as sensory-motor blockade scores, pain scores, and other convertible parameters used throughout nerve block in future trials. Third, this study did not assess the quality of individual RCTs in detail and identified the potential characteristics associated with FI. It is noteworthy that there is no clear cutoff point for FI. A higher FI appears to have a positive relationship with a larger sample size; however, this does not necessarily indicate a rigorous RCT.

## Conclusions

Although the incidence of HDP may be statistically significant based on the reported *P*-value, we found that the statistically significant difference in RCTs after brachial plexus blocks is often fragile, and the findings may, in large part, depend on a small number of events. Therefore, when evaluating the findings of trials, FI should be used as an intuitive aid for interpreting RCTs results. Given the robustness of the results of clinical trials, further trials with larger sample size are needed.

## Data Availability

The datasets used and/or analysed during the current study are available from the corresponding author on reasonable request.

## Abbreviations

RCTs: Randomized Clinical Trials
HDP: Hemidiaphragmatic Paralysis
FI: Fragility Index
FQ: Fragility Quotient
IQR: Inter-quartile Range
